# Neglected Tropical Diseases among the Association of Southeast Asian Nations (ASEAN): Overview and Update

**DOI:** 10.1371/journal.pntd.0003575

**Published:** 2015-04-16

**Authors:** Peter J. Hotez, Maria Elena Bottazzi, Ulrich Strych, Li-Yen Chang, Yvonne A. L. Lim, Maureen M. Goodenow, Sazaly AbuBakar

**Affiliations:** 1 Sabin Vaccine Institute and Texas Children’s Hospital Center for Vaccine Development, Departments of Pediatrics and Molecular Virology and Microbiology, National School of Tropical Medicine, Baylor College of Medicine, Houston, Texas, United States of America; 2 Department of Biology, Baylor University, Waco, Texas, United States of America; 3 James A. Baker III Institute for Public Policy, Rice University, Houston, Texas, United States of America; 4 Tropical Infectious Diseases Research and Education Centre, Department of Medical Microbiology, University of Malaya, Kuala Lumpur, Malaysia; 5 Department of Parasitology, University of Malaya, Kuala Lumpur, Malaysia; 6 Department of Pathology, Immunology, and Laboratory Medicine, University of Florida, College of Medicine, Gainesville, Florida, United States of America; University of Queensland, AUSTRALIA

## Abstract

The ten member states of the Association of Southeast Asian Nations (ASEAN) constitute an economic powerhouse, yet these countries also harbor a mostly hidden burden of poverty and neglected tropical diseases (NTDs). Almost 200 million people live in extreme poverty in ASEAN countries, mostly in the low or lower middle-income countries of Indonesia, the Philippines, Myanmar, Viet Nam, and Cambodia, and many of them are affected by at least one NTD. However, NTDs are prevalent even among upper middle-income ASEAN countries such as Malaysia and Thailand, especially among the indigenous populations. The three major intestinal helminth infections are the most common NTDs; each helminthiasis is associated with approximately 100 million infections in the region. In addition, more than 10 million people suffer from either liver or intestinal fluke infections, as well as schistosomiasis and lymphatic filariasis (LF). Intestinal protozoan infections are widespread, while leishmaniasis has emerged in Thailand, and zoonotic malaria (*Plasmodium knowlesi* infection) causes severe morbidity in Malaysia. Melioidosis has emerged as an important bacterial NTD, as have selected rickettsial infections, and leptospirosis. Leprosy, yaws, and trachoma are still endemic in focal areas. Almost 70 million cases of dengue fever occur annually in ASEAN countries, such that this arboviral infection is now one of the most common and economically important NTDs in the region. A number of other arboviral and zoonotic viral infections have also emerged, including Japanese encephalitis; tick-borne viral infections; Nipah virus, a zoonosis present in fruit bats; and enterovirus 71 infection. There are urgent needs to expand surveillance activities in ASEAN countries, as well as to ensure mass drug administration is provided to populations at risk for intestinal helminth and fluke infections, LF, trachoma, and yaws. An ASEAN Network for Drugs, Diagnostics, Vaccines, and Traditional Medicines Innovation provides a policy framework for the development of new control and elimination tools. Together with prominent research institutions and universities, the World Health Organization (WHO), and its regional offices, these organizations could implement important public health improvements through NTD control and elimination in the coming decade.

## Introduction

**Fig 1:**
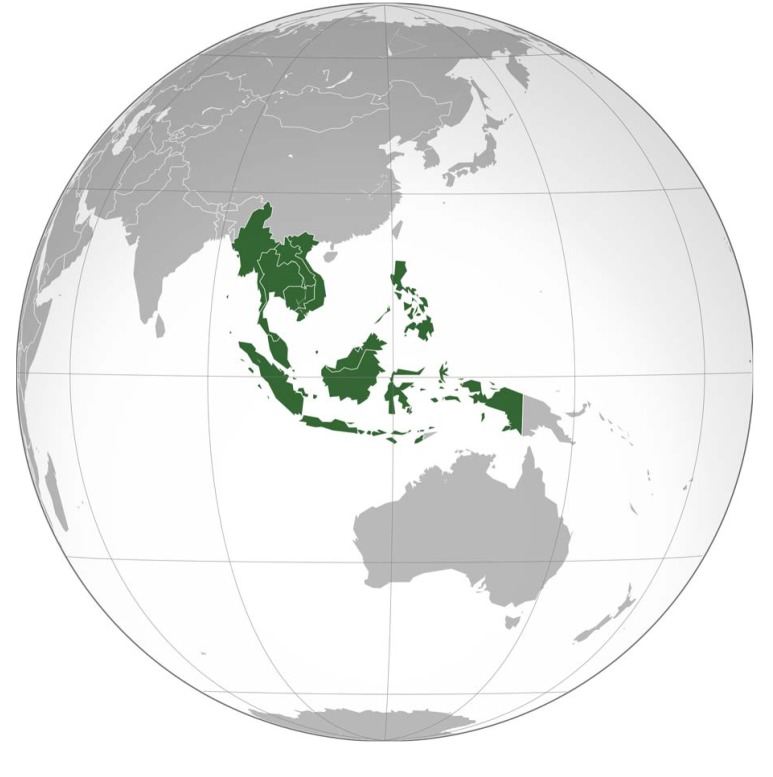
The Association of Southeast Asian Nations (ASEAN). Image Credit: Wikimedia contributor Addicted04.

ASEAN was founded in 1967 in order to promote economic and cultural development; to promote trade, agricultural, industrial, and scientific collaboration; and to promote peace and stability in the region [1]. Today, ten member states—Brunei Darussalam, Cambodia, Indonesia, Lao People’s Democratic Republic (PDR), Malaysia, Myanmar, the Philippines, Singapore, Thailand, and Viet Nam—comprise ASEAN (Fig 1) and, together, include a population of approximately 636 million people or almost 10% of the global population (Table 1) [2,3]. The largest number of people live in Indonesia (>250 million people), followed by the Philippines and Viet Nam (with approximately 107 and 93 million people, respectively) [2].

**Table 1:** The Countries of ASEAN.

Country	Population(July 2014) [2]	% <US$1.25 per day [5,101]	% <US$2 per day or below poverty line [2,6]	Total <US$2 per day or below poverty line*
Brunei Darussalam	0.4 million	Not reported	Not reported	Not reported
Cambodia	15.4 million	18.6% (2009)	49.5% (2009)	7.6 million
Indonesia	253.6 million	16.2% (2011)	43.3% (2011)	109.8 million
Lao PDR	6.8 million	37.4% (2010)	22% (2013)	1.5 million
Malaysia	30.1 million	0% (2009)	2.3% (2009)	0.7 million
Myanmar	55.7 million	Not reported	32.7% (2007)	18.2 million
Philippines	107.7 million	18.4% (2009)	41.5% (2009)	44.7 million
Singapore	5.6 million	Not reported	Not reported	Not reported
Thailand	67.7 million	0.4% (2010)	4.1% (2010)	2.8 million
Viet Nam	93.4 million	8.3% (2010)	11.3% (2012)	10.6 million
ASEAN	636.4 million			195.9 million
Global	7.2 billion [102]			2.8 billion [7]
% of Global Population	8.8%			7.0%

*Calculated by multiplying the proportion of people living below the different poverty levels by the total population

The countries that comprise ASEAN have experienced impressive economic growth in recent years. According to the Organization of Economic Cooperation and Development (OECD), the six major ASEAN economies—Indonesia, Malaysia, the Philippines, Singapore, Thailand, and Viet Nam—have averaged between 4.6% and 7.1% economic growth since 2011 (and as projected into 2015) [4]. However, such rapid growth has also left a substantial fraction of people economically marginalized. Overall, almost 200 million people in ASEAN countries, or roughly 30% of the population, live in extreme poverty, i.e., on less than US$2 per day, or below their national poverty lines [5–7]. Together, Indonesia and the Philippines account for about three-quarters of ASEAN’s poor, although even middle-income nations, such as Malaysia and Thailand, have hundreds of thousands of economically disadvantaged people.

Neglected tropical diseases (NTDs) mostly affect people who live in extreme poverty; these diseases are also known to promote poverty even further because of their chronic and debilitating effects [8,9]. Indeed, a substantial portion of the “bottom 200 million” people of the Southeast Asian region suffers from more than one NTD. While the NTDs of Southeast Asia were reviewed in 2010 [10,11], here we summarize some of the revised estimates published over the last four years for the 14 of 17 NTDs (as defined by WHO) endemic among the countries of ASEAN (Box 1). These estimates are based on publicly-available and updated WHO Preventive Chemotherapy and Transmission (PCT) data, supplemented with additional data, in order to provide an overview and suggest policy recommendations for the region.

**Box 1. NTDs affecting ASEAN countries from World Health Organization’s List of 17 Neglected Tropical Diseases**

Helminths

- Cysticercosis/Taeniasis*
- Dracunculiasis (guinea-worm disease)
- Echinococcosis*
- Foodborne trematodiases*
- Lymphatic filariasis*
- Onchocerciasis (river blindness)
- Schistosomiasis*
- Soil-transmitted helminthiases*

Protozoa

- Chagas disease
- Human African trypanosomiasis (sleeping sickness)
- Leishmaniasis*

Bacteria

- Buruli ulcer*
- Leprosy (Hansen’s disease)*
- Trachoma*
- Yaws*

Virus

- Dengue/Severe dengue*
- Rabies*

*Major NTDs affecting ASEAN Countries

Additional Major NTDs Affecting ASEAN Countries

Strongyloidiasis and Toxocariasis

Intestinal Protozoan Infections

Malaria caused by *Plasmodium knowlesi*

Melioidosis

Japanese Encephalitis

Nipah Virus Infection

Enterovirus 71

(From http://www.who.int/neglected_diseases/diseases/en/, accessed July 20, 2014)

## Neglected Helminth Infections

New studies by Pullan et al. [12] on soil-transmitted helminth infections determined that 126.7 million people in Southeast Asia are infected with *Ascaris* roundworms, while 115.3 million are infected with *Trichuris* whipworms, and 77.0 million have hookworm infections, including a high proportion of cases with *Ancylostoma ceylanicum* infections, a unique zoonotic hookworm infection found in ASEAN countries, especially Malaysia, Thailand, Cambodia, and Lao PDR [13,14]. Thus, approximately one-half of the people of Southeast Asia living in poverty have one or more soil-transmitted helminth infection. Indigenous populations, such as the Orang Asli communities in peninsular Malaysia, are disproportionately affected, with high levels of community prevalence and intensities [15]. Hookworm infections in the region have long been known as a leading cause of anemia [16–18], and, moreover, helminthic infections with *Trichuris trichiura* or *Ascaris lumbricoides* in the region are also statistically associated with anemia and iron deficiency anemia, leading to incidences of low-birth-weight infants and inadequate growth and mental development in children, as well as high maternal mortality and low productivity in adults [8]. Two additional soil-transmitted helminth infections—strongyloidiasis and toxocariasis—are common among indigenous and other populations living in poverty [19,20] and are probably widespread in the region, but there are no published data on their overall prevalence. According to WHO and its PCT database, almost 120 million pre-school- and school-aged children require periodic deworming for soil-transmitted helminth infections in ASEAN, which accounts for more than 13% of the global population eligible for deworming (Table 2) [21,22]. Similarly, ASEAN countries account for more than 13% of the global population that require mass treatment for lymphatic filariasis (LF) [23,24], or more than 15 million people if we extrapolate from the widely used number of 120 million people globally living with LF [25]. It is very encouraging, though, that through the Global Program to Eliminate LF (GPELF), launched in 2000, there now is a clear path to the elimination of LF. Within ASEAN, Viet Nam, Cambodia, and Malaysia have already achieved their targets [26,27], with the other member states making excellent progress [27].

**Table 2:** NTDs of the ASEAN Countries according to WHO PCT data and other sources.

Country	Children with intestinal helminth infections in 2012 [20,21]	Population requiring treatment for lymphatic filariasis in 2012 [22,23]	Population requiring treatment for schistosomiasis annually in 2013 [28,33]	New Leprosy cases reported in 2012 [103]	Dengue fever apparent and inapparent cases [73]
Brunei Darussalam	Not reported	15,000		2	12,732 apparent
					38,421 inapparent
					<0.1 million total
Cambodia	1.2 million preSAC	Under surveillance	6,008	475	0.4 million apparent
	2.9 million SAC				1.2 million inapparent
					1.6 million total
Indonesia	16.9 million preSAC	113.2 million	3,035	18,994	7.6 million apparent
	43.5 million SAC				23.0 million inapparent
					30.6 million total
Lao PDR	0.5 million preSAC	132,644	9,164	88	0.1 million apparent
	1.4 million SAC				0.4 million inapparent
					0.5 million total
Malaysia	<0.1 million				
Myanmar	3.2 million preSAC	41.7 million		3,013	1 million apparent
	8.1 million SAC				3.0 million inapparent
					4.0 million total
Philippines	8.9 million preSAC	29.4 million	499,901	2,150	3.1 million apparent
	22.2 million SAC				9.3 million inapparent
					12.4 million total
Singapore	None			15	0.2 million apparent
					0.5 million inapparent
					0.7 million total
Thailand	<0.1 million	73,495		220	1.9 million apparent
					1.8 million inapparent
					7.7 million total
Viet Nam	3.4 million preSAC	Under surveillance		296	2.6 million apparent
	5.2 million SAC				8.0 million inapparent
					10.6 million total
ASEAN	34.1 million preSAC	184.5 million	517,796	25,253	68.2 million
	83.3 million SAC				
	117.4 million total				
Global	875.9 million	1.41 billion	237 million	232,857	390 million
%Global Population	13.4%	13.1%	0.2%	10.8%	17.4%

SAC: school-age children, preSAC: pre-school age children.

Among the platyhelminth infections, Furst et al. [28] estimate that 9.3 million people suffer from bile duct liver fluke infections in the region (39% of the global number of cases), including 8.03 million cases of opisthorchiasis, mostly in Lao PDR and Thailand, and 1.25 million cases of clonorchiasis in Viet Nam. Approximately 3.4 million people live with intestinal flukes in the Philippines and Thailand, which represents more than half the global disease burden [28]. It is worth emphasizing that the full effect of these fluke infections may go beyond the primary disease symptoms. For example, there is some evidence that fluke infections, which constitute a strong risk factor for bile duct cancer (cholangiocarcinoma) [29], have the greatest incidence rates in Thailand (85/100,000 versus <0.5/100,000 in the Western world) [29–31]. In addition, according to WHO, in the Philippines in 2013, about 500,000 school-aged children required preventive chemotherapy and over 865,000 people were treated for schistosomiasis caused by *Schistosoma japonicum*, with a prevalence as high as 65% in some communities [32–34]. In addition, WHO reports, over 150,000 people were treated in Cambodia and Lao PDR for what were likely *S*. *mekongi* infections, and a little over 10,000 were treated in Indonesia for infections with, likely, *S*. *japonicum* [33]. Another parasite involving gastropod intermediate hosts in its life cycle and endemic to the region is *Angiostrongylus*, which causes eosinophilic meningitis, eosinophilic meningoencephalitis, and, rarely, ocular disease during occasional outbreaks in the region, particularly in Thailand. Due do difficulties in diagnosis of the actual parasite, the true number of infections in the past might be underestimated [35]. Taeniasis and cysticercosis, common cestode diseases [36,37], and possibly echinococcosis [38], are likewise found in Southeast Asia, but there is a dearth of information regarding their prevalence and disease burden.

## Neglected Protozoan Infections

None of the protozoan kinetoplastid infections on WHO’s list of 17 NTDs are considered highly endemic to Southeast Asia. However, visceral leishmaniasis, caused by *Leishmania siamensis*, has recently emerged in southern Thailand [39], with the first case of cutaneous leishmaniasis in Thailand reported in 2012 [40]. Intestinal protozoan infections are widespread among Southeast Asia’s most impoverished populations, including indigenous populations [41]. The major intestinal protozoan infections are giardiasis and cryptosporidiosis, which have been linked to malnutrition or impaired growth in Southeast Asia [42,43], and *Blastocystis* infection [44]. In recent reports from the region, *B*. *hominis*, in particular, has gained increased attention for causing opportunistic infections in individuals immunocompromised by cancer or HIV-1 infection [45–48]. The intestinal protozoa have emerged as important waterborne parasites in Southeast Asia [49,50]. Toxoplasmosis is also common among the Orang Asli indigenous communities of Malaysia [51], and presumably other indigenous and impoverished populations across the region, although no overall prevalence estimates for either the intestinal protozoan infections or toxoplasmosis are published.

While not ordinarily classified as an NTD, Southeast Asia faces a serious disease burden from malaria, with both *Plasmodium falciparum* and *P*. *vivax* found in every ASEAN country [52]. An estimated 7.5 million infections occur in ASEAN countries [53,54]. *P*. *knowlesi* has emerged as the fifth (and zoonotic) human malaria species, with a large human focus first reported in 2004 from Sarawak, Malaysian Borneo, and, subsequently, elsewhere in Peninsular Malaysia and Southeast Asia (except Lao PDR) [55–57]. Today, *P*. *knowlesi* malaria is found in wild monkey populations, where it is the most common cause of clinical and severe malaria in Malaysia [56]. There are no overall published prevalence estimates for this type of malaria in Southeast Asia, but by defining actual and potential monkey reservoirs, Moyes et al. [56] recently provided innovative maps of transmission areas, and defined areas of intense transmission to Malaysia and other areas.

## Neglected Bacterial Infections

Tuberculosis (TB) is also not classified as an NTD, although WHO has determined that a high disease burden of TB occurs in six ASEAN countries—Cambodia, Indonesia, Myanmar, the Philippines, Thailand, and Viet Nam [58]. Cases of extremely drug-resistant TB have occurred in Thailand and Malaysia [58,59]. Leprosy and trachoma are the only major bacterial NTDs from WHO’s list that are found commonly in Southeast Asia. Buruli ulcer and yaws also occur in Southeast Asia, but are not as widely prevalent as in sub-Saharan Africa and Australia [10,60,61], although yaws is still endemic in parts of Indonesia [61]. WHO estimates that ASEAN countries account for approximately 10% of the world’s registered leprosy cases, with three-quarters of the cases found in Indonesia [62]. Trachoma is still considered endemic only in Cambodia and Lao PDR, and surveillance for elimination is underway in Viet Nam [63]. A recent analysis of febrile illnesses in the Mekong region identified scrub typhus (*Orientia tsutsugamushi*), murine typhus (*Rickettsia typhi*), and members of the spotted fever group rickettsiae as important etiologies, as are leptospirosis (*Leptospira* spp.), salmonellosis (*Salmonella enterica* serovar Typhi and Paratyphi), and melioidosis (*Burkholderia pseudomallei*) [64,65]. Indeed, melioidosis has emerged as a major neglected bacterial infection and serious cause of gram-negative sepsis and bacteremic pneumonia in Southeast Asia, but especially in northern Thailand [66–68]. While the number of cases may have increased in Indonesia following the 2004 Asian tsunami [66], there are no recent published incidence or disease burden estimates. Leptospirosis, on the other hand, is an emerging zoonotic bacterial infection causing high numbers of infections with death following natural disasters, especially flooding in densely populated urban areas [69,70], such as in the aftermath of Typhoon Ondoy in the Philippines in 2009 [71]. Leptospirosis is also on the rise in Malaysia, with a markedly increased number of deaths in more recently reported incidents [72].

## Neglected Viral Infections

Dengue (and other arboviral infections) and rabies are the two major viral NTDs. New estimates by Bhatt et al. [73] indicate that approximately 68 million apparent and inapparent dengue infections occur annually in the countries of ASEAN, accounting for more than 17% of the global disease burden. Moreover, dengue represents a major economic threat, with some estimates indicating that the disease results in almost US$1 billion in annual economic losses for the region [74]. Other important arboviral infections include Japanese encephalitis (JE), West Nile virus, and chikungunya [75,76]. Less described, but potentially endemic, infections include those caused by Zika virus, Tembusu virus, and Usutu virus [77,78]. Tick-borne diseases, such as tick-borne encephalitis and Crimean-Congo hemorrhagic fever, are also potentially emerging neglected viral diseases, particularly amongst the economically marginalized population. There is a dearth of published studies describing the incidence and prevalence of tick-borne diseases in countries of ASEAN, although these diseases are endemic in neighboring countries such as Japan and China [79,80]. Still, other key emerging viral infections include Nipah virus, a zoonosis from fruit bats, which was transmitted to pigs and humans [81], or enterovirus 71, an important cause of Hand-Foot-and-Mouth disease and meningoencephalitis in children [82]. There is a need for more active surveillance of these diseases and estimates of incidence and disease burden for the Southeast Asian region. Currently, all of the JE affected countries of Southeast Asia, except for Indonesia, maintain sentinel, subnational, or national surveillance programs, while Cambodia, Thailand, and Viet Nam implement programs of vaccination [83]. The global burden of disease of four arbovirus infections, including JE and chikungunya, was recently reported, although the burden affecting the Southeast Asian region as a whole was not specified [84]. Finally, canine rabies represents an important public health threat to the region. While canine rabies has been practically eliminated in Singapore and Malaysia [85], the remainder of the region, especially the poorest areas of countries such as Indonesia or the Philippines, continue to report cases [86].

## Establishing a Public Policy and Framework for the Region

A summary of the number of cases of the major NTDs affecting ASEAN is shown in Table 3. The major soil-transmitted helminth infections cause the most frequent NTDs, followed by dengue, LF, liver/lung fluke infections, malaria, schistosomiasis, and leprosy. However, there is an extreme dearth of information for the intestinal protozoan and other infections, zoonotic malaria from *P*. *knowlesi*, toxoplasmosis, all of the bacterial NTDs other than leprosy, and all of the viral NTDs other than dengue fever. Only fragmentary information is available from local settings, with no national and/or regional burdens comprehensively assessed. This calls for urgent action to conduct active surveillance for these NTDs and to define the full extent of NTD-caused illnesses within the respective countries and collectively within the region. Efforts incorporating advanced tools, such as remote sensing or geographic information systems, as was initiated for the soil-transmitted helminth infections [87], should be generally implemented. In addition, there is a need to intensify and improve assessments of the economic impact of the NTDs among ASEAN countries, especially amongst the economically marginalized populations. This concerted effort will equip the region with evidence-based information, which is pertinent, especially as ASEAN moves closer towards her aspiration to become a single entity as the ASEAN Economic Community (AEC) in 2015. Educating cabinet officials and parliamentarians in these nations on how NTDs are trapping 200 million Southeast Asians in poverty, while exercising a “whole of government” approach, could create opportunities for advocacy to bring in new and additional resources to fight these diseases.

**Table 3:** Summary of the major NTDs and malaria in the ASEAN countries.

Disease	Number of cases Southeast Asian Region[in millions]	Population infected / [%]	Global Burden / [%]	reference
Ascariasis	126.7	21	15.5	[12]
Trichuriasis	115.3	19	24.7	[12]
Hookworm infection	77.0	13	17.5	[12]
Dengue fever	68.2	11	17.4	[73]
Lymphatic filariasis	Not determined	2	13.1	[25]
Liver fluke infection	9.3	1	39.2	[28]
Malaria	7.5	1	3.6	[53,54]
Intestinal fluke infection	3.4	<1	50.7	[28]
Schistosomiasis	1.0	<1	<1	[33,34]
Leprosy	0.02	<1	<1	[103]

Through integrated mass drug administration, there are needs to expand coverage for the soil-transmitted helminth infections, LF, schistosomiasis, and trachoma. Currently, the United States Agency for International Development (USAID) through its Neglected Diseases Program is supporting integrated mass drug administration for these diseases in the nations of Cambodia, Indonesia, Lao PDR, the Philippines, and Viet Nam [88]. Given how widespread the intestinal protozoa are in ASEAN, it might be worthwhile investigating whether nitazoxanide is effective and safe if added to current drug regimens [89,90]. Together with intersectoral collaboration that includes water, sanitation, and hygiene (WASH) [91], there may be opportunities for multilateral collaboration on the control and elimination of these diseases. Such scientific cooperation will be particularly important for the vector-borne NTDs, including the arboviruses and other emerging viral infections, which are transmitted across national borders and are not currently amenable to mass drug administration (MDA) approaches.

For many of the NTDs affecting the region though, new drugs, diagnostics, and vaccines are needed. Therefore, there is dire urgency to identify mechanisms for support of the development of such products even though return on investment may be through poverty reductions rather than more traditional mechanisms. Meaningful outcomes can be generated with active involvement of universities and biotechnology enterprises, which have the capabilities for translational research and development, especially in some of the major research institutes in Singapore, Malaysia, Thailand, and elsewhere, as well as through developing country vaccine manufacturers in Thailand, Indonesia, and Viet Nam. These activities must include efforts to engage innovative financing schemes through organizations such as the Asian Development Bank or the Malaysian Investment Development Authority (MIDA), among others. Great benefits have been achieved in the past through collaborative efforts such as the Regional Network on Asian Schistosomiasis and Other Important Zoonosis (RNAS+), founded in 1998. By strengthening the collaboration between control and research authorities in the region (and including China), RNAS+ now provides advice on regional strategies on the mobilization of resources with respect to multinational projects on several parasitic diseases in Southeast Asia, primarily schistosomiasis but also other helminth infections [92,93]. A new, promising development is the establishment of the ASEAN Network for Drugs, Diagnostics, Vaccines, and Traditional Medicines Innovation (ASEAN-NDI) [94], founded in 2009 to parallel the African Network for Drugs and Diagnostics Innovation (ANDI), a network championed by the World Health Organization Special Programme for Research and Training in Tropical Diseases (WHO-TDR) [95]. These initiatives helped to implement the idea of establishing regional innovation networks. Activities with the network include the assessment of the product research and development (R&D) landscape for the triple burden of disease in the region: infectious tropical diseases, non-communicable diseases, and preventable diseases due to accidents and traumas. The results of the mapping exercise indicated a diverse spectrum of capacity for ASEAN member states to pursue R&D on drugs, diagnostics, vaccines, and traditional medicine [96]. Likewise, the geographically broader Asia Pacific NTD Initiative [97] in support of WHO’s Regional Strategic Plan for Integrated NTD Control in the South-East Asia Region [98] and WHO’s Regional Action Plan for Neglected Tropical Diseases in the Western Pacific [99] assists in the formulation of national NTD plans of action, monitoring programs, and the mobilization of internal and external funds. The stated goal of the five-year (2012–2016) Asia Pacific NTD Initiative is to reduce suffering and increase productivity through minimal investments in capacity-building, health education, integrated planning, and technical assistance to overcome the existing logistical bottlenecks. While regional governments and other donors have already contributed nearly 50% of the total budget, the initiative still reports a US$121 million dollar funding gap. Nonetheless, various countries have already started to see benefits from this initiative. For instance, the WHO target of deworming at least 75% of school-age children has been reached in Cambodia, Lao PDR, and Viet Nam [100]. The continued funding of such innovative partnerships, including setting aside a specific percentage of the Gross Domestic Product (GDP) for the purpose, will be essential for future successes. Finally, science diplomacy is potentially also in the remit of ASEAN, and this area could be repurposed to focus on codevelopment of vaccines, drugs, and diagnostics for NTDs now affecting Southeast Asia [66].

**Key Learning Points**

- Almost 200 million people live in extreme poverty in the ten ASEAN countries.
- Helminth infections are the most common NTD, especially the intestinal helminthiases and fluke infections.
- Intestinal protozoan infections are widespread and *Plasmodium knowlesi* infection is an important cause of severe malaria.
- Vector-borne bacterial and viral infections are major causes of morbidity and mortality in the region, as are Nipah virus and enterovirus 71 infection.
- An ASEAN Network for Drugs, Diagnostics, Vaccines, and Traditional Medicines Innovation (ASEAN-NDI) provides a policy framework for the development of new control and elimination tools.

**Top Five Papers**

1. Ngui R, Lim YA, Chong Kin L, Sek Chuen C, Jaffar S. Association between anaemia, iron deficiency anaemia, neglected parasitic infections and socioeconomic factors in rural children of West Malaysia. PLoS neglected tropical diseases. 2012;6(3):e1550.
2. Furst T, Keiser J, Utzinger J. Global burden of human food-borne trematodiasis: a systematic review and meta-analysis. The Lancet Infectious diseases. 2012;12(3):210–21.
3. Moyes CL, Henry AJ, Golding N, Huang Z, Singh B, Baird JK, et al. Defining the geographical range of the Plasmodium knowlesi reservoir. PLoS neglected tropical diseases. 2014;8(3):e2780.
4. Limmathurotsakul D, Dance DA, Wuthiekanun V, Kaestli M, Mayo M, Warner J, et al. Systematic review and consensus guidelines for environmental sampling of Burkholderia pseudomallei. PLoS neglected tropical diseases. 2013;7(3):e2105.
5. Shepard DS, Undurraga EA, Halasa YA. Economic and disease burden of dengue in Southeast Asia. PLoS neglected tropical diseases. 2013;7(2):e2055.
